# An acoustic dataset for surface roughness estimation in milling process

**DOI:** 10.1016/j.dib.2024.111108

**Published:** 2024-11-04

**Authors:** N.R. Sakthivel, Josmin Cherian, Binoy B Nair, Abburu Sahasransu, L.N.V. Pratap Aratipamula, Singamsetty Anish Gupta

**Affiliations:** a Department of Mechanical Engineering, Amrita School of Engineering, Amrita Vishwa Vidyapeetham, Coimbatore, India; b Department of Electronics and Communication Engineering, Amrita School of Engineering, Amrita Vishwa Vidyapeetham, Coimbatore, India

**Keywords:** Condition monitoring, Machine learning, Machining, Acoustic, Milling

## Abstract

Machining process involves numerous variables that can influence the desired outcomes, with surface roughness being a critical quality index for machined products. Surface roughness is often a technical requirement for mechanical products as it can lead to chatter and impact the functional performance of parts, especially those in contact with other materials. Therefore, predicting surface roughness is essential. This dataset comprises 7444 audio files containing acoustic signal samples recorded using a 44.1 kHz microphone during the milling of mild steel with a tungsten carbide tool on a BFW YF1 vertical milling machine. Various combinations of speed, feed and depth of cut were used, and surface roughness values measured using a Carl Zeiss E-35B profile-meter are provided for each combination. Additionally, an example workflow indicating the possible use of the data to estimate the surface roughness from the acoustic signals is presented. This dataset is the first publicly available resource for surface roughness measurement using sound signals in milling, offering significant potential for reuse in related research and applications.

Specifications Table

**Table:** 

Subject	Manufacturing Engineering, Applied Machine Learning.
Specific subject area	Surface Roughness estimation in milling of Mild Steel*.*
Type of data	*.*au (Audio files of milling operation with specific feed, speed and depth of cut), .xlsx (an excel file mentioning the roughness values for different combinations of feed, speed and depth of cut.
Data collection	Data was collected using a BFW YF1 vertical milling machine, with mild steel workpieces (8 mm × 20 mm × 8 mm) machined using tungsten carbide inserts. An acoustic sensor (microphone) was used to measure the acoustic emissions from the milling process. Experiments are carried out using different combinations of spindle speed (500 rpm, 1000 rpm), feed (5 mm,10 mm), and depth of cut (1 mm, 0.75 mm,0.5 mm, and 0.25 mm), with surface roughness (Ra) measured to categorize signals into chatter or good. A total of 320 sets of data were conducted across different cutting parameters*.*
Data source location	Amrita School of Engineering, Coimbatore, Amrita Vishwa Vidyapeetham, India*.*
Data accessibility	Repository name: Mendeley Data Data identification number: DOI: 10.17632/rrhmxdj988.1 Direct URL to data: https://data.mendeley.com/datasets/rrhmxdj988/1 The data publicly available at Mendeley Data. The dataset can be directly downloaded and the sensor data as well as measured roughness values will be available. The files associated with this dataset are licensed under a Creative Commons Attribution 4.0 International license
Related research article	Not Applicable*.*

## 1 Value of the Data

• Online Surface Roughness Measurement and Monitoring: The data enable real-time assessment and monitoring of surface roughness in milling processes. Measuring the surface roughness of a workpiece is critical in manufacturing. This is because a smoother surface makes the final product work better, last longer, and resist wear. This data helps in optimizing machining parameters to improve product quality and reduce manufacturing defects, thus enhancing the efficiency of industrial processes.
• Online Chatter Detection: Chatter is a self-excited type vibration that can arise in the milling process at specific combinations of speed, feed and depth of cut. This makes the surface rougher, also roughing the tool faster. By providing comprehensive acoustic signal data, this dataset facilitates the early detection of chatter. Researchers can use this data to develop and refine algorithms for chatter prediction and suppression, leading to more stable and reliable machining processes.
• Accelerating Innovation in Machining: The dataset allows researchers and product developers to bypass the need for extensive experimental setups, saving significant time and resources. Access to this data can expedite the development and testing of new machining strategies and tools, fostering innovation in the field.

## 2 Background

The primary motivation behind compiling this dataset is to investigate the impact of milling process parameters on surface roughness and chatter, and to understand how these factors affect the quality of a machined product. Milling is a widely used manufacturing process where the quality of the final product is highly dependent on the surface finish. Surface roughness is a critical parameter as it influences the mechanical properties, wear resistance, and aesthetic appearance of the machined components. High surface roughness can lead to poor performance, reduced fatigue life, and increased friction, which are undesirable in precision engineering applications. In addition, chatter, a form of self-excited vibration occurring during the milling process, significantly affects surface quality. It can lead to increased surface roughness, dimensional inaccuracies, and can even damage the cutting tool. Understanding the relationship between process parameters and their effect on surface roughness and chatter is essential for optimizing milling operations and ensuring high-quality output*.*

## 3 Data Description

### 3.1 Data orientation

**Fig. 1:**
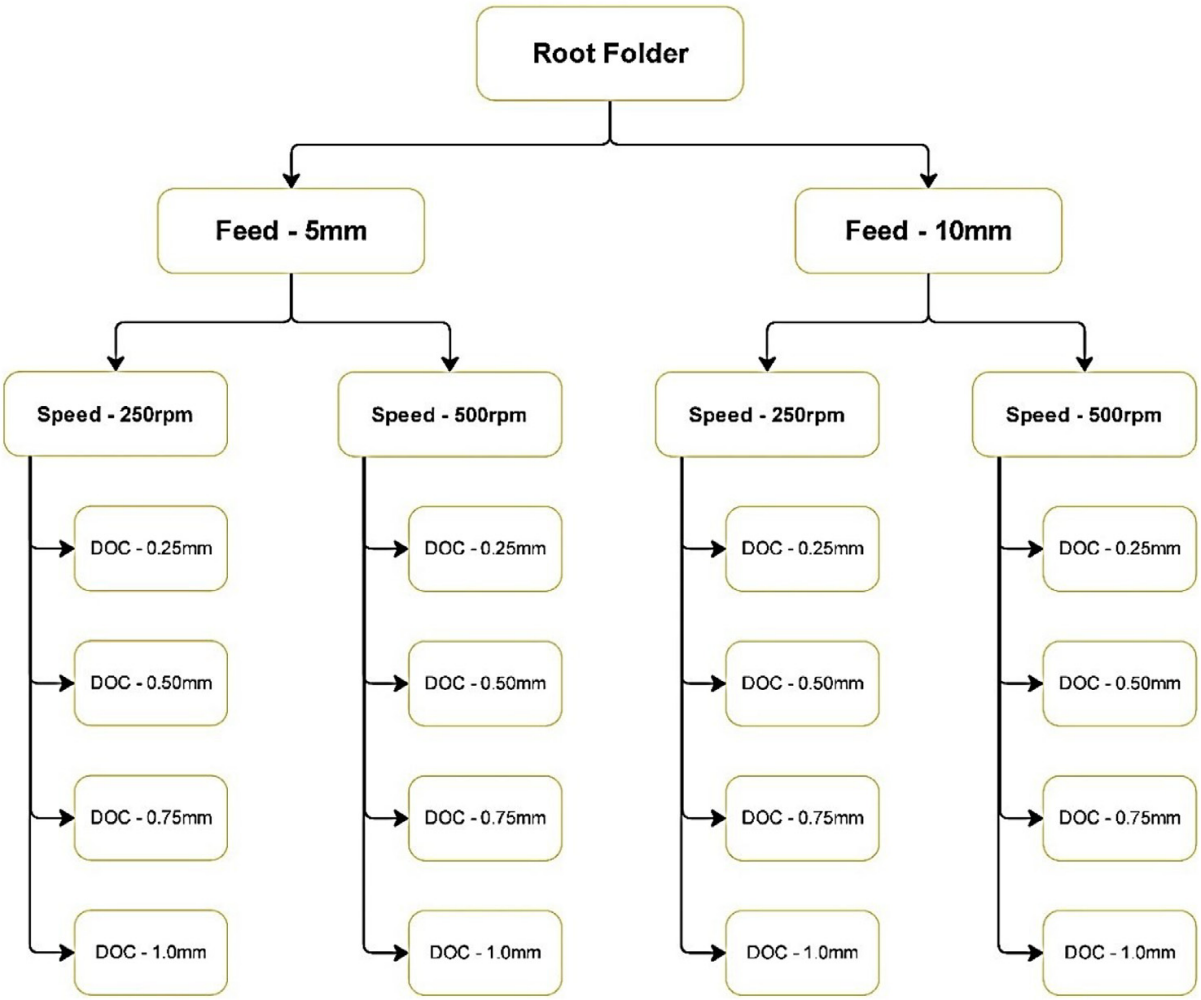
Visual representation of data orientation.

The dataset comprises 7444 audio files (in .au format) collected using a microphone with 44.1 KHz sampling rate for various combinations of feed, speed, and depth of cut in milling, organized into different subfolders. The dataset also contains a separate file (in .xlsx format) listing the combinations of feed, speed, depth of cut along with the surface roughness values calculated using a profilometer (Roughness parameters measured are described in Table 1 and the specifications of the profilometer are given in Table 2). The dataset is organized as given in Fig. 1 below.

**Table 1:** Measured surface roughness parameters.

Abbreviation	Expansion	Abbreviation	Expansion
Ra	Average Roughness	Pc	Peak count
Rz	Mean Peak-to-valley Height	Pt	Total profile height
Rzmax	Maximum Peak-to-valley Height	Rmr	Material Ratio of the profile
RSm	Mean width of profile elements	Rk	Core roughness depth
Rq	Root mean square roughness	Rpk	Reduced peak height
Rp	Maximum profile peak height	Rvk	Reduced Valley Depth
Rt	Total Height of the Roughness profile	Mr1	Material ratio at Peak level
R3z	Average Maximum height of the	Mr2	Material ratio at valley
	Profile		Level
Vo	Oil Retention Volume	K	Kurtosis of the surface profile

**Table 2:** Specifications of Profilometer.

Parameter	Specification
Make	CARL ZEISS
Model	E-35B
Measuring Range	Z Axis ± 160 µm, X Axis 12.5 mm
Cut-off value	0.08 mm, 0.25 mm, 0.8 mm, 2.5 mm
Resolution	0.01 µm
Tracing speed	0.6 mm/s
Probing Force	4 mN
Material of stylus	Diamond
Radius of stylus	5µm

As shown in Fig. 1, the Root- folder contains two subfolders: “feed-5mm” (feed of 5 mm) and “feed-10 mm” (feed of 10 mm). Each feed subfolder is further divided into two subfolders representing different speeds: “speed-500 rpm” (speed of 500 rpm) and “speed-250 rpm” (speed of 250 rpm). Inside these speed subfolders, there are four additional subfolders corresponding to different depths of cut: “doc-1.0 mm” (depth of cut 1.0 mm), “doc-0.75 mm” (depth of cut 0.75 mm), “doc-0.5 mm” (depth of cut 0.5 mm), and “doc-0.25 mm” (depth of cut 0.25 mm). Each of these subfolders contains folders with audio signal files for the chosen combination of speed, feed, and depth of cut. While the duration of the audio files varies, they all share the same sampling frequency of 44,100 Hz. For each such combination above, the surface roughness of the workpiece is also measured.

Table 1 below lists the surface roughness measurements made by the Carl-Zeiss E-35B profilometer for each speed-feed-depth of cut combination.

The specifications of the profilometer used for surface roughness measurement are given in Table 2 below.

### 3.2 Data summary statistics

Statistical parameters such as Mean, Median, Maximum, Range, Skewness, Kurtosis and Standard Deviation of the acoustic signals belonging to different machining parameter combinations of speed, feed and depth of cut (DoC) as illustrated in Fig. 1 have been computed to provide a better understanding of the dataset and are presented in Table 3.

**Table 3:** Summary statistics of the dataset.

Feed	5 mm		10 mm		
Speed	250 rpm	500 rpm	250 rpm	500 rpm	
DoC					

0.25 mm	Mean	−1.79E-05	−1.69E-05	−1.68E-05	−1.8E-05
	Median	−61.0E-05	−15E-05	−12E-05	−18E-05
	Maximum	0.999	0.999	0.999	0.999
	Range	1.999	1.999	1.999	1.999
	Skewness	0.029	0.016	0.005	0.007
	Kurtosis	3.147	3.271	3.263	3.102
	Std Dev.	0.112	0.114	0.091	0.102

0.5 mm	Mean	−1.45E-05	−1.27E-05	−8.17E-06	−1.45E-05
	Median	−61.03E-05	−9.16E-05	6.10E-05	−54.9E-05
	Maximum	0.999	0.999	0.999	0.999
	Range	1.99	1.999	1.999	1.999
	Skewness	0.022	0.013	−0.005	0.031
	Kurtosis	2.953	3.532	3.194	3.382
	Std Dev.	0.125	0.119	0.102	0.121

0.75 mm	Mean	−1.59E-05	−1.51E-05	−12.6E-05	−1.30E-05
	Median	−21.3E-05	−18.3E-05	3.05E-05	−51.8E-05
	Maximum	0.999	0.999	0.999	0.999
	Range	1.999	1.999	1.999	1.999
	Skewness	0.007	0.0125	0.008	0.075
	Kurtosis	3.018	3.764	3.346	5.306
	Std Dev.	0.123	0.123	0.111	0.127

1.0 mm	Mean	−32.2E-05	−49E-05	−14.2E-05	−15E-05
	Median	−109.8E-05	−604.2E-05	−30.5E-05	−335E-05
	Maximum	0.999	0.999	0.999	0.999
	Range	1.999	1.999	1.999	1.999
	Skewness	0.024	0.0691	0.007	0.048
	Kurtosis	3.249	3.063	3.151	3.112
	Std Dev.	0.323	0.333	0.344	0.329

## 4 Data

### 4.1 Visualization

(a) *Histogram Plots*

**Fig. 2:**
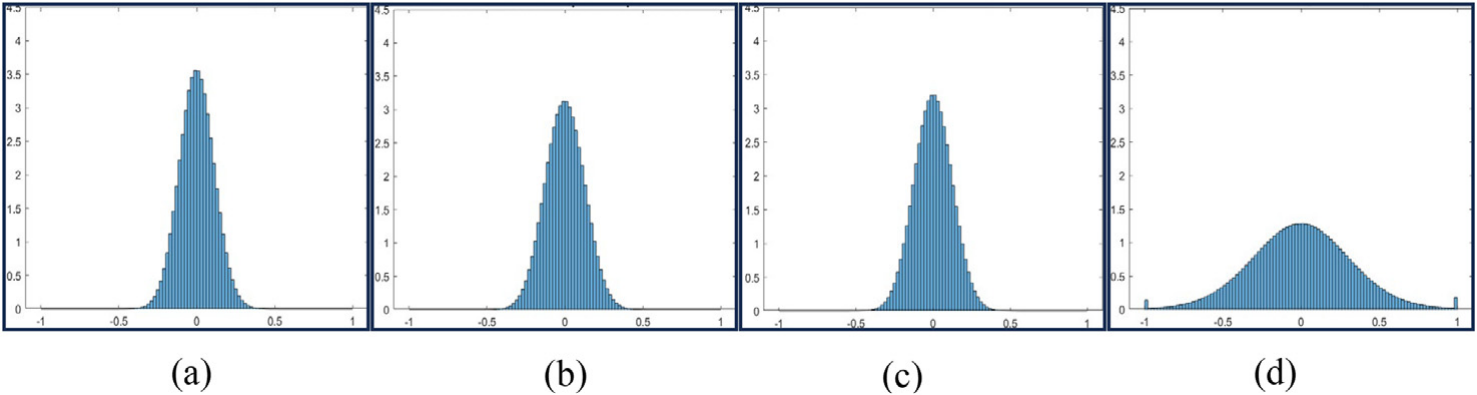
Histogram Plot for Feed = 5 mm, Speed = 250 rpm and DoC = 0.25 mm(a), 0.5 mm(b), 0.75 mm(c), and 1 mm(d).

**Fig. 3:**
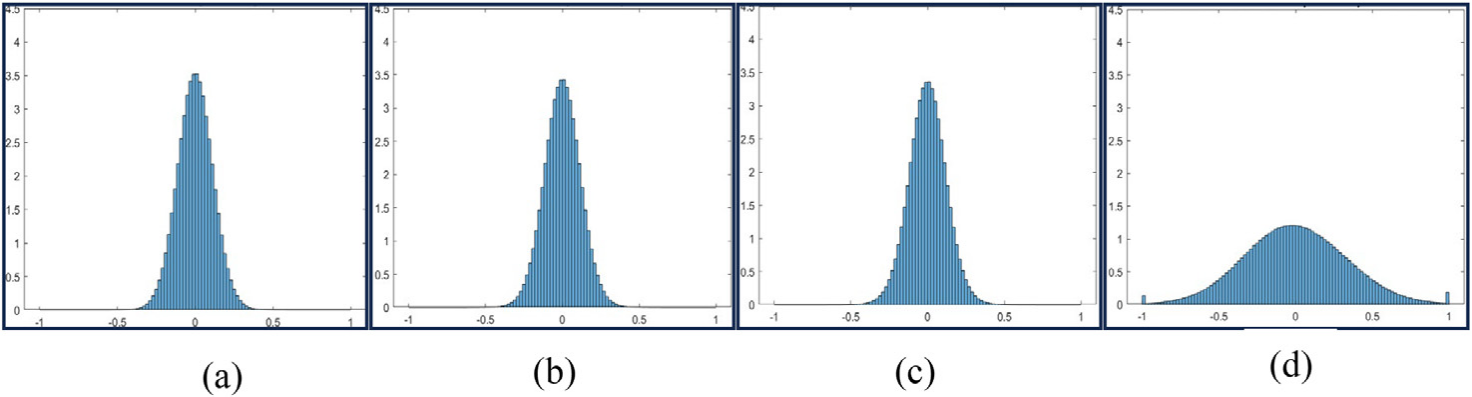
Histogram Plot for Feed = 5 mm, Speed = 500 rpm and DoC = 0.25 mm (a), 0.5 mm (b), 0.75 mm (c), and 1 mm (d).

**Fig. 4:**
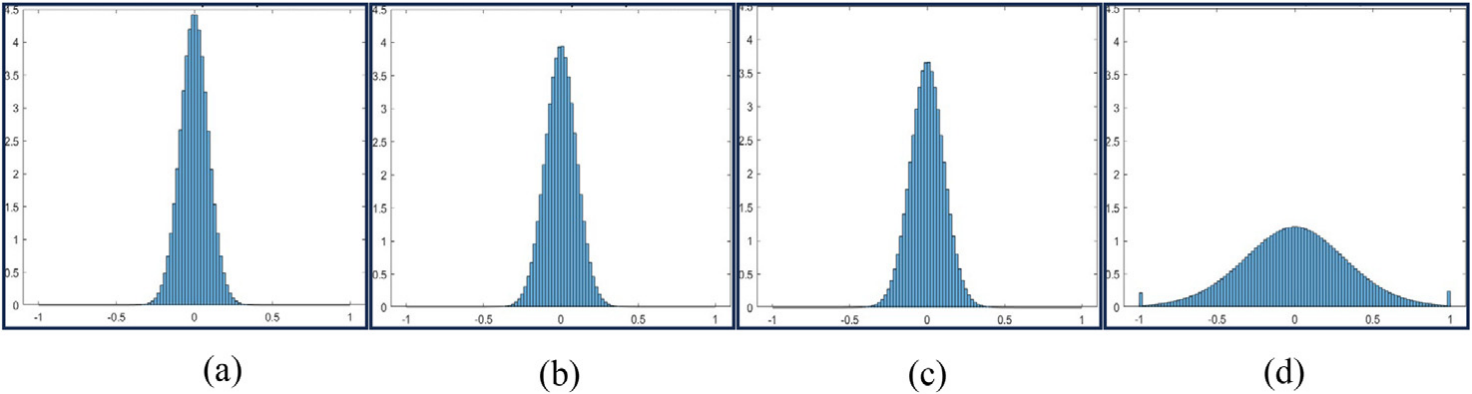
Histogram Plot for Feed = 10 mm, Speed = 250 rpm and DoC = 0.25 mm (a), 0.5 mm (b), 0.75 mm (c), and 1mm(d).

**Fig. 5:**
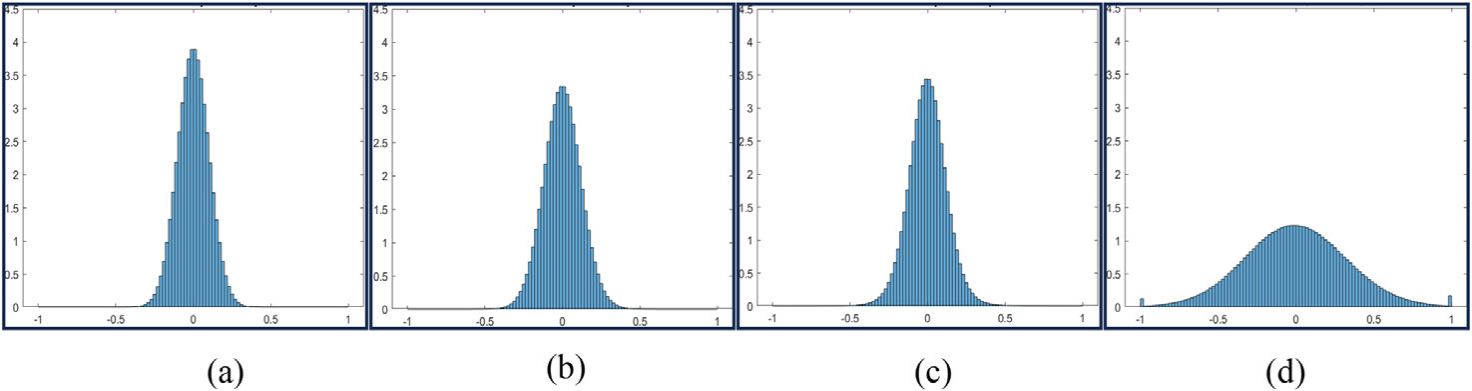
Histogram Plot for Feed = 10 mm, Speed = 500 rpm and DoC = 0.25 mm (a), 0.5 mm (b), 0.75 mm (c), and 1 mm (d).

Histograms of data collected for all combinations of cutting parameters are plotted (number of bins: 100) as shown in Fig. 2, Fig. 3, Fig. 4, Fig. 5. This helps in understanding the impact of different machine settings on the acoustic signals.

(b) *Time domain representation*

**Fig. 6:**
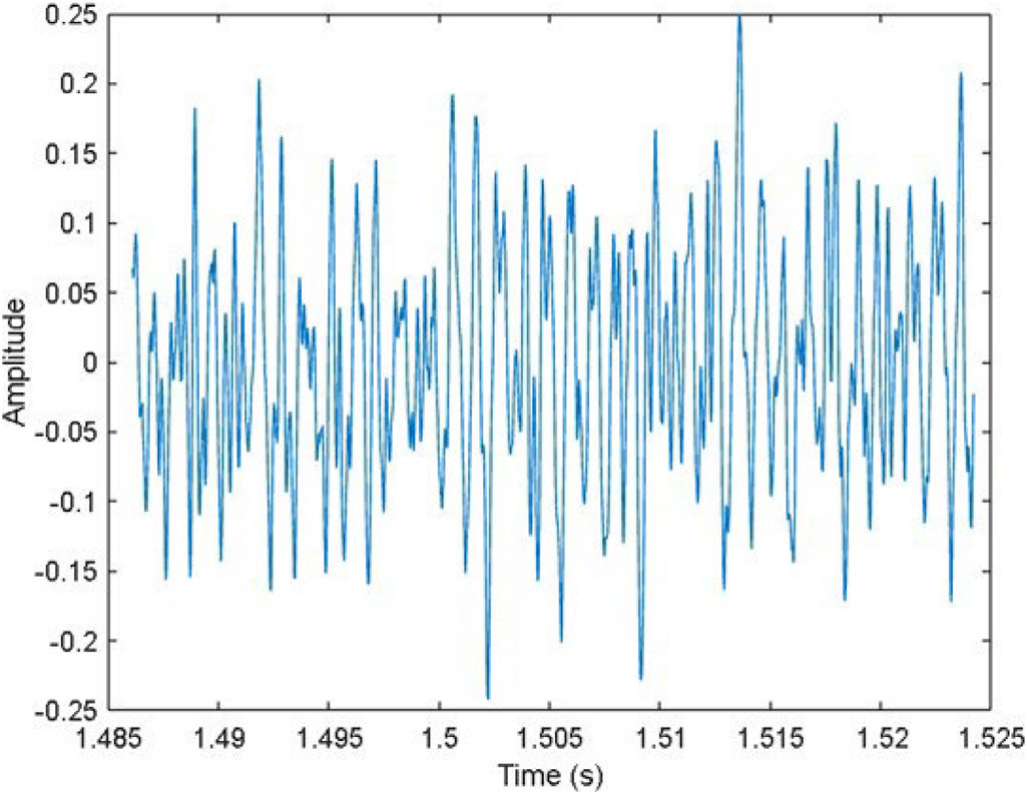
Time domain plot of the signal acquired by acoustic sensor.

To gain deeper understanding of the dynamic behavior occurring during the milling process, we have included a plot of an audio sample in the time domain in Fig. 6 for better visualization. This plot visualizes how the signal's amplitude fluctuates over time. Analyzing this time domain plot alongside the measured surface roughness value can provide valuable insights on tool wear and the presence of chatter.

(c) *Frequency Domain Representation*

**Fig. 7:**
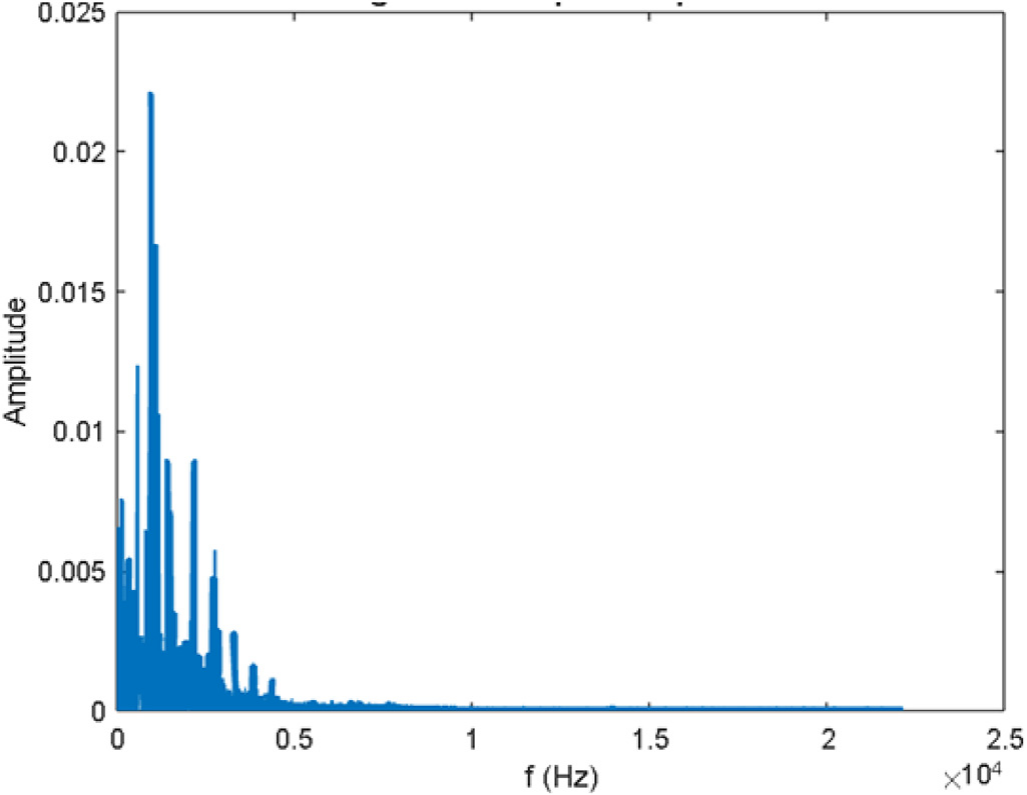
Single-sided amplitude spectrum.

For a better understanding of the data samples, the amplitude spectrum derived from Fast Fourier Transform (FFT) is presented in Fig. 7. This visualization helps gain insight into the behavior of signal in the frequency domain, which reveals a different perspective compared to the raw audio data over time.

FFT is an efficient algorithm for computing the Discrete Fourier Transform (DFT). The DFT converts time-domain signal into its frequency domain representation, revealing the specific frequencies that compose the signal. Unlike the direct computation of DFT, which requires *N*^2^ operations for N data points, FFT reduces this complexity to *N* log_2_ *N*, making it vastly more efficient. The mathematical formula for the DFT of a sequence *x*(*n*) of length N is:

$$X\left(k\right)=\sum_{n=0}^{N-1}x\left(n\right)e^{-\frac{i2\pi \text{kn}}{N}}$$

Where X(k) is the kth frequency component of the DFT, x(n) is the nth time-domain sample and N is the total number of time-domain samples. The amplitude spectrum is a simplified version of the FFT output. While FFT provides more technical details, the amplitude spectrum focuses on a more straightforward aspect of how much energy is present at each frequency in the signal, highlighting the most prominent frequencies. The amplitude spectrum is of two types: single-sided and Two-sided, while Two-sided amplitude spectrum displays amplitudefrom positive and negative frequencies separately which is crucial for phase-sensitive analysis, single- sided is used with real signals, displays only positive amplitude up to Nyquist frequency. Fig. 7 depicts the single-sided amplitude spectrum of an audio sample.

### 4.2 Suggested methodology for surface roughness identification

Machine learning based methods are increasingly becoming popular for applications such as milling [1,2,7,8],turning [3,4], boring [5,6] and drilling grinding [9,10] However, the applicability of acoustic signals for monitoring of surface roughness in milling process has not been explored in the literature so far.

**Fig. 8:**
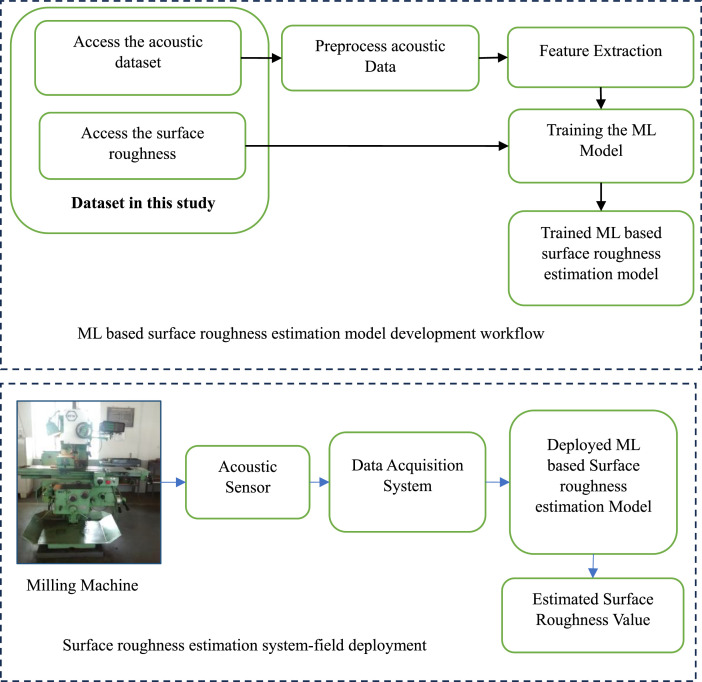
Suggested methodology for ML based surface roughness estimation system.

The suggested methodology for estimation of surface roughness using Machine Learning (ML) based models is given in Fig. 8 below. The ML model for surface roughness can be trained offline using the dataset provided here. Once the optimal ML model has been developed, it can be deployed in the field to estimate the surface roughness based on the acoustic signals acquired from the milling process.

### 4.3 Methodology illustration using a baseline ML based surface roughness prediction system

A Baseline Model for predicting the surface roughness (Ra) based on acoustic features has been built to demonstrate the effectiveness of the workflow presented above. Two features are extracted from the normalized data, namely, short time energy and pitch.

Short time energy:

Short time energy is a temporal feature which captures variations in signal intensity over time. It is computed by squaring the amplitude of the signal over a short window of time and then summing these squared values.

$$E_{r}=\frac{1}{N}\sum_{n=1}^{N}{\left|x_{r}\left(n\right)\right|}^{2}$$

Where, $E_{r}$ is the short time energy of the r-th frame, N is the number of samples in the current frame, $x_{r}\left(n\right)$ is the amplitude of the audio signal at the nth sample within the r-th frame.

*Pitch:*

It is the fundamental perceptual attribute of sound that relates to the fundamental frequency of a periodic sound wave. The fundamental frequency $f_{0}$ is the lowest frequency component of a complex sound and determines the perceived pitch. Mathematically,

$$\text{Pitch},P=f_{0}=\frac{1}{T}$$

Where, T is the period of the sound wave. Sound wave can be described as a sinusoidal function of time. A sinusoidal wave is mathematically expressed as:

$$x\left(t\right)=\text{Acos}\left(2\pi f_{0}t+\emptyset \right))$$

Or

$$x\left(t\right)=\text{Asin}\left(2\pi f_{0}t+\emptyset \right)$$

Where, A is the amplitude of the wave, t is time and ∅ is the phase of the wave.

Pitch detection algorithms use various methods to estimate $f_{0}$ from audio signals. Common techniques include Fourier transform (FT) for spectral analysis and autocorrelation methods. Once $f_{0}$ is estimated, it directly corresponds to the perceived pitch P of the sound.

The pitch and short time energy values were used as input features to the baseline model which is a simple multiple-linear regression model. The surface roughness value Ra is set as the target variable. The dataset was split with 80 % used for training and the remaining 20 % reserved for testing. Ordinary least squares method was used to train the model. In general, for a linear regression model, regression equation is given as:

$$y=a_{0}+a_{1}x_{1}+a_{2}x_{2}+a_{3}x_{3}\ldots \ldots \ldots a_{n}x_{n}$$

Where, $a_{1},a_{2},a_{3},\ldots a_{n}$ are coefficients of the respective features $x_{1},x_{2},x_{3}\ldots ..x_{n}$. and a_0_ is the intercept.

The Regression equation derived from the data using simple ordinary least-squares regression is as follows:

$$\text{Ra}=0.00567P+0.048E_{r}$$

Where, P is the Pitch and E_r_ is the Short-time Energy.

**Fig. 9:**
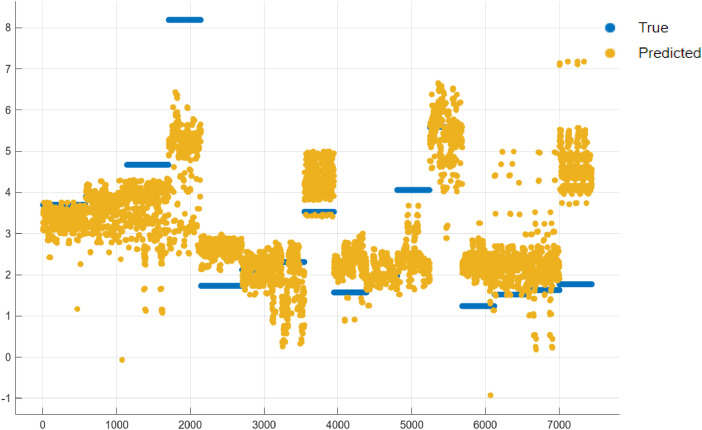
Actual (True) vs predicted Ra for the baseline model.

Model performance was evaluated using three performance metrics: R^2^ (R-Squared), Mean Squared Error(MSE) and Root Mean Squared Error (RMSE). The results are tabulated in Table 4. The response plot of Actual vs Predicted Ra values is presented in Fig. 9.

**Table 4:** Performance metrics of the Baseline model.

Evaluation Metric	Training	Testing
R-Squared	0.43	0.38
MSE	1.85	1.96
RMSE	1.36	1.4

It can be seen that the baseline model is able to estimate surface roughness values, however, the R^2^ values and the MSE, RMSE values that there is a significant scope for improvement in the prediction accuracy. More advanced ML/ Deep Learning models are expected to offer much better performance.

## 5 Experimental Design, Materials and Methods

**Fig. 10:**
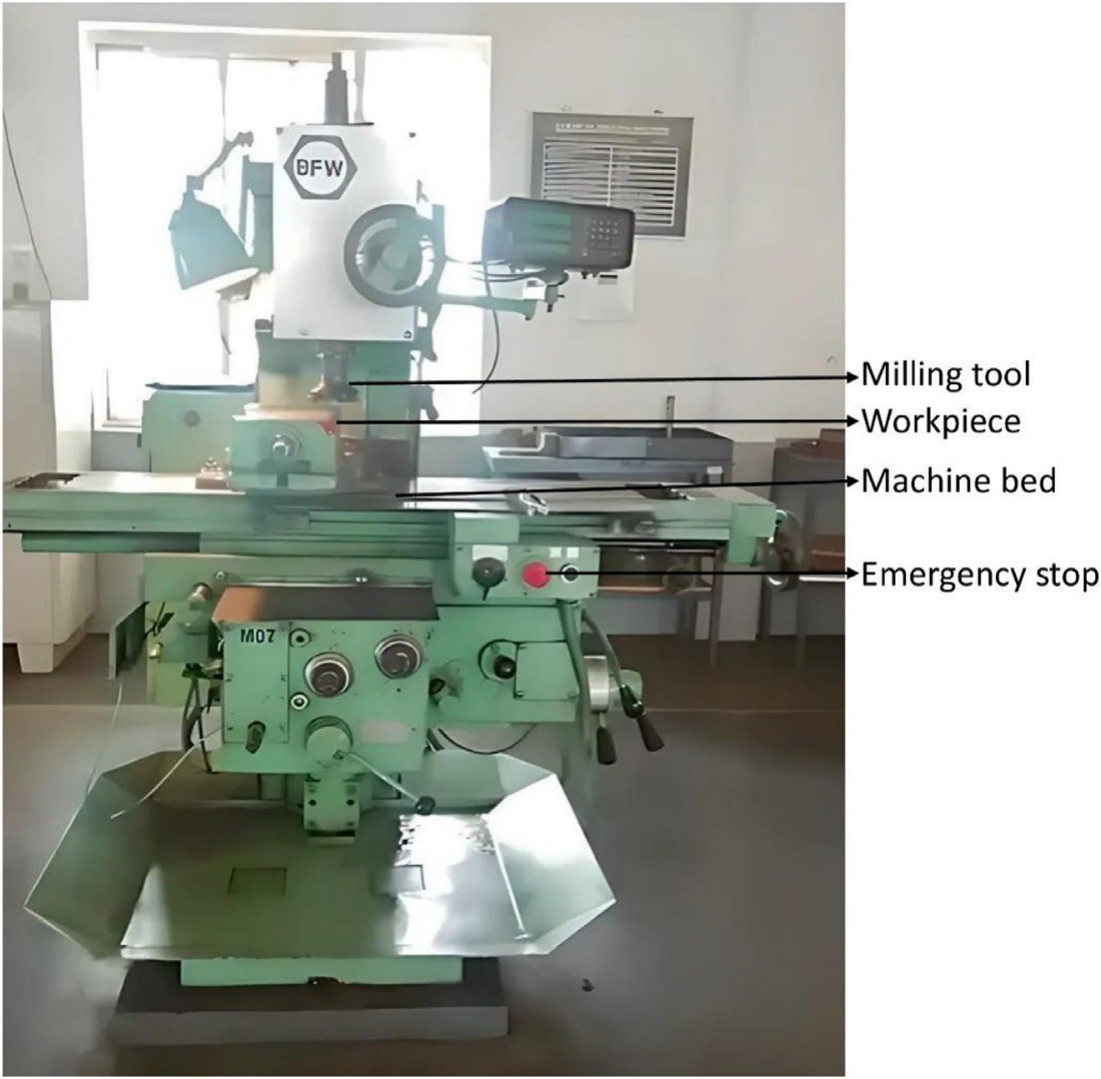
Vertical milling machine (BFW-Model YF1) used in the experiment.

In this experiment, a vertical milling machine (BFW model YF1) is used. The machine is shown in Fig. 10. The material used for machining is mild steel of dimension (8 mm × 20 mm × 8 mm). In the milling cutter inserts of type Tungsten Carbide is used. In vertical milling machine, spindle spins in a vertical orientation over the table. A 44.1 kHz microphone is used to acquire the acoustic signals. These signals are stored in the computer memory. The signals can then be processed to extract different features and estimate surface roughness*.*

**Fig. 11:**
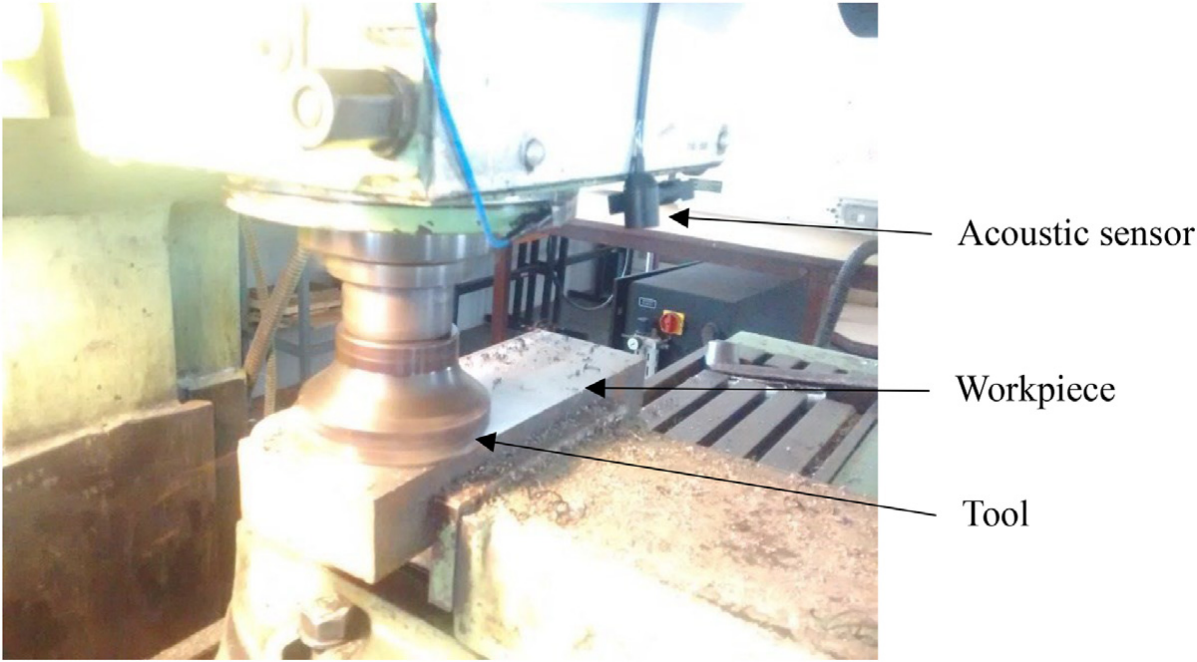
Positioning of acoustic sensor on the milling machine.

The positioning of the acoustic sensor on the milling machine is given in Fig. 11 below.

The acoustic signals are acquired from the vertical milling machine for different spindle speed, feed and depth of cut. For each speed, speed and depth of cut the surface roughness of the machine surface are measured. Total of 320 experiments with four depth of cuts, two feeds and two speeds. The specifications of the milling machine are given in Table 5. A Carl-Zeiss E-35B profilometer is used to measure the surface roughness values of the workpiece for each feed-speed-dept of cut combination. The complete dataset can be downloaded from [7].

**Table 5:** Vertical milling machine specification (BFW- Model YF1).

Parameter	Value
Table Clamping Area	1245 × 230 mm
Number of spindle speeds	12 steps 45–2000 rpm
Number of speeds	18 steps 16–800 mm/min
Spindle motor	4 HP/ 1420 rpm
Feed Motor	0.75 HP/ 1440 rpm
Machine weight	1800 kg

The specifications of the microphone used to acquire the dataset are given in Table 6 below.

**Table 6:** Specifications of microphone used for Data Acquisition.

Parameter	Value
Frequency Response	20 Hz – 20 kHz
Sampling Rate	44.1 kHz
Sensitivity	35 dBV/Pa
Dynamic Range	120 dB
Signal-to-Noise Ratio	75 dB
Impedance	150 ohms
Build Quality	Metal casting, shock-resistant mount

### 5.1 Setup challenges

The Experiments were carried out in a controlled, quiet environment to minimize any potential sources of external noise that could interfere with the accuracy of the collected audio data. By eliminating background noise and ensuring consistent acoustic conditions, we were able to capture high-quality audio signals that accurately reflect the milling process. This approach was crucial for preserving the integrity of the data, allowing for more reliable analysis and interpretation of the sound characteristics associated with milling operations. Careful attention was also given to equipment setup and environmental factors (no abnormal weather events or power fluctuations were observed during the acquisition process) to further ensure the clarity and precision of the recorded acoustic data.

## Limitations

The microphone is used in capturing acoustic data in the experiment with a sampling rate of 44.1 kHz, which might not be enough to capture all the higher frequency details in milling. This can lead to the circumstance where some of the required data for accurate surface roughness prediction are lost. For example, high frequency vibrations arising due to the cutter-work interaction which are critical to detect tool wear or onset of chatter problem might get aliased or lost.

## Ethics Statement

The authors have read and followed the ethical requirements for publication in Data in Brief and confirm that the current work does not involve human subjects, animal experiments, or any data collected from social media platforms.

## Credit Author Statement

**N R Sakthivel:** Conceptualization, Methodology, data logging. **Josmin Cherian:** Data logging. **Binoy B Nair:** Software, Visualization, Writing- original draft preparation. **Abburu Sahasransu:** Writing- Reviewing and Editing. **L.N.V. Pratap Aratipamula:** Writing- Reviewing and Editing, **Singamsetty Anish Gupta:** Data curation.

## Data Availability

Mendeley DataMilling Surface Roughness Acoustic Sensor Dataset (Original data).
